# A second-order dynamical approach with variable damping to nonconvex smooth minimization

**DOI:** 10.1080/00036811.2018.1495330

**Published:** 2018-07-09

**Authors:** Radu Ioan Boţ, Ernö Robert Csetnek, Szilárd Csaba László

**Affiliations:** a Faculty of Mathematics, University of Vienna, Vienna, Austria; b Department of Mathematics, Technical University of Cluj-Napoca, Cluj-Napoca, Romania

**Keywords:** Boris Mordukhovich, Second-order dynamical system, nonconvex optimization, Kurdyka–Łojasiewicz inequality, convergence rate, 90C26, 90C30, 65K10

## Abstract

We investigate a second-order dynamical system with variable damping in connection with the minimization of a nonconvex differentiable function. The dynamical system is formulated in the spirit of the differential equation which models Nesterov's accelerated convex gradient method. We show that the generated trajectory converges to a critical point, if a regularization of the objective function satisfies the Kurdyka- Lojasiewicz property. We also provide convergence rates for the trajectory formulated in terms of the Lojasiewicz exponent.

## 1. Introduction

Consider the (not necessarily convex) optimization problem

(1) $$\underset{x\in R^{n}}{\inf}g(x),$$

where $g:R^{n}⟶R$ is a Fréchet differentiable function with $L_{g}$-Lipschitz continuous gradient, i.e. there exists $L_{g}\geq 0$ such that $∥\nabla g(x)-\nabla g(y)∥\leq L_{g}∥x-y∥$ for all $x,y\in R^{n}$. We associate to (1) the second-order dynamical system (for $t\geq t_{0}$)

(2) $$\begin{array}{r} \begin{array}{rl} & \ddot{x}(t)+\left(\frac{\alpha }{t}+\gamma \right)\dot{x}(t)+\nabla g(x(t))=0,\\ & x(t_{0})=u_{0},\;\dot{x}(t_{0})=v_{0}, \end{array} \end{array}$$

where $t_{0}>0,u_{0},v_{0}\in R^{n},\alpha \in R$ and γ∈(0,+∞).

The study of the system (2) is motivated by the recent developments related to the approaching of the solving of convex optimization problems from a continuous perspective.

In [1], Su, Boyd and Candes proposed the following dynamical system

(3) $$\begin{array}{r} \begin{array}{rl} & \ddot{x}(t)+\frac{\alpha }{t}\dot{x}(t)+\nabla g(x(t))=0,\\ & x(t_{0})=u_{0},\;\dot{x}(t_{0})=v_{0}, \end{array} \end{array}$$

as the continuous counterpart of the Nesterov's accelerated gradient method [see [2]] for minimizing *g* in the convex case. This research has been deepened by Attouch and his co-authors [see [3,4]], who proved that, if α>3, then the generated trajectory x(t) converges to a minimizer of *g* as t→+∞, while the convergence rate of the objective function along the trajectory is $o(1/t^{2})$. The convergence of the trajectory is actually the continuous counterpart of a result due to Chambolle and Dossal [see [5]], which proves the convergence of the iterates of the modified FISTA algorithm [see [6]].

Recently, in [7], investigations have been performed concerning the convergence rate of the objective function along the trajectory in the subcritical case α≤3, while some open questions related to the asymptotic properties of the trajectory have been formulated.

In this manuscript, we carry out, in the nonconvex setting, an asymptotic analysis of the dynamical system (2), which can be seen as a perturbation of the dynamical system (3) that models Nesterov's accelerated gradient method in the convex case. To the best of our knowledge, this is the first contribution addressing second-order dynamical systems with variable damping associated to nonconvex optimization problems. We show that the generated trajectory converges to a critical point of *g* as t→+∞, provided the following regularization of *g*,

$$H:R^{n}\times R^{n}⟶R,\;H(u,v)=g(u)+\frac{1}{2}∥u-v{∥}^{2},$$

satisfies the Kurdyka–Łojasiewicz inequality. Moreover, we derive convergence rates in the terms of Łojasiewicz exponent, for the trajectory, its velocity and its acceleration. One of the major future goals is to study the asymptotic properties of the system (2) in case γ=0. For other investigations of the asymptotic analysis of second-order dynamical systems with time-dependent damping, we refer to the papers of Haraux and Jendoubi [8] and Balti [9].

For α=0, the convergence of the trajectory generated by (2) to a critical point of *g* has been shown by Bégout, Bolte and Jendoubi in [10] in the hypothesis that *g* is of class $C^{2}$ and it satisfies the Kurdyka–Łojasiewicz property with a desingularizing function satisfying a restrictive condition [see also the papers of Haraux and Jendoubi [11] and Chill and Jendoubi [12]]. On the other hand, the dynamical system (2) is, for α=0, a particular instance of the second-order dynamical system of proximal-gradient type studied in [13].

The following numerical scheme, with starting points $x_{0},x_{1}\in R^{n}$,

(4) $$(\forall k\geq 1)\;\left\{\begin{array}{l} y_{k}=x_{k}+\frac{(1-\gamma \sqrt{s})k-\alpha \gamma \sqrt{s}}{k+\alpha }(x_{k}-x_{k-1}),\\ x_{k+1}=y_{k}-s\nabla g(y_{k}), \end{array}\right.$$

where $s\leq 1/L_{g}$ is the step size, can be seen as a discrete counterpart of (2). One can notice that for γ=0, this iterative scheme algorithm is similar to Nesterov's accelerated convex gradient method.

In the following, we prove that (2) can be seen in an informal way as the exact limit of (4)). We take to this end in (4) small step sizes and follow the same approach as Su, Boyd and Candes in [1, Section 2]. For this purpose, we rewrite (4) in the form

(5) $$\frac{x_{k+1}-x_{k}}{\sqrt{s}}=\frac{(1-\gamma \sqrt{s})k-\alpha \gamma \sqrt{s}}{k+\alpha }\cdot \frac{x_{k}-x_{k-1}}{\sqrt{s}}-\sqrt{s}\nabla g(y_{k})\;\forall \;k\geq 1$$

and introduce the *Ansatz* $x_{k}\approx x(k\sqrt{s})$ for some twice continuously differentiable function $x:[0,+\infty )\to R^{n}$. We let $k=t/\sqrt{s}$ and get $x(t)\approx x_{k},\;x(t+\sqrt{s})\approx x_{k+1},\;x(t-\sqrt{s})\approx x_{k-1}.$ Then, as the step size *s* goes to zero, from the Taylor expansion of *x*, we obtain

$$\frac{x_{k+1}-x_{k}}{\sqrt{s}}=\dot{x}(t)+\frac{1}{2}\ddot{x}(t)\sqrt{s}+o(\sqrt{s})$$

and

$$\frac{x_{k}-x_{k-1}}{\sqrt{s}}=\dot{x}(t)-\frac{1}{2}\ddot{x}(t)\sqrt{s}+o(\sqrt{s}).$$

Furthermore, since

$$\sqrt{s}∥\nabla g(y_{k})-\nabla g(x_{k})∥\leq \sqrt{s}L_{g}∥y_{k}-x_{k}∥=\sqrt{s}L_{g}\left|\frac{(1-\gamma \sqrt{s})k-\alpha \gamma \sqrt{s}}{k+\alpha }\right|∥x_{k}-x_{k-1}∥=o(\sqrt{s}),$$

it follows $\sqrt{s}\nabla g(y_{k})=\sqrt{s}\nabla g(x_{k})+o(\sqrt{s})$. Consequently, (5) can be written as

$$\begin{array}{rl} \dot{x}(t)+\frac{1}{2}\ddot{x}(t)\sqrt{s}+o(\sqrt{s}) & =\frac{(1-\gamma \sqrt{s})t-\alpha \gamma s}{t+\alpha \sqrt{s}}\left(\dot{x}(t)-\frac{1}{2}\ddot{x}(t)\sqrt{s}+o(\sqrt{s})\right)\\ & \;-\sqrt{s}\nabla g(x(t))+o(\sqrt{s}) \end{array}$$

or, equivalently

$$\begin{array}{rl} \left(t+\alpha \sqrt{s})(\dot{x}(t)+\frac{1}{2}\ddot{x}(t)\sqrt{s}+o(\sqrt{s})\right) & =((1-\gamma \sqrt{s})t-\alpha \gamma s)(\dot{x}(t)-\frac{1}{2}\ddot{x}(t)\sqrt{s}+o(\sqrt{s}))\\ & \;-\sqrt{s}(t+\alpha \sqrt{s})\nabla g(x(t))+o(\sqrt{s}). \end{array}$$

Hence,

$$\frac{1}{2}(2t+\alpha \sqrt{s}-\gamma t\sqrt{s}-\alpha \gamma s)\ddot{x}(t)\sqrt{s}+(\gamma t\sqrt{s}+\alpha \sqrt{s}+\alpha \gamma s)\dot{x}(t)+\sqrt{s}(t+\alpha \sqrt{s})\nabla g(x(t))\;=o(\sqrt{s}).$$

After dividing by $\sqrt{s}$ and letting s→0, we obtain

$$t\ddot{x}(t)+(\gamma t+\alpha )\dot{x}(t)+t\nabla g(x(t))=0,$$

which, after division by *t*, gives (2), namely

$$\ddot{x}(t)+\left(\frac{\alpha }{t}+\gamma \right)\dot{x}(t)+\nabla g(x(t))=0.$$

## 2. Existence and uniqueness of the trajectory

We consider on the finite-dimensional space $R^{n}$ the Euclidean topology. If $x\in R^{n}$ is a local minimizer of *g*, then ∇g(x)=0. We denote by

$$\mathrm{crit}(g)=\{x\in R^{n}:\nabla g(x)=0\}$$

the set of critical points of *g*.

We are considering in the asymptotic analysis of the dynamical system (2) strong global solutions.

Definition 2.1 We say that $x:[t_{0},+\infty )\to R^{n}$ is a strong global solution of (2), if the following properties are satisfied:

1. $x,\dot{x}:[t_{0},+\infty )\to R^{n}$ are locally absolutely continuous, in other words, absolutely continuous on each interval $\left[t_{0},T\right]$ for $t_{0}<T<+\infty$;
2. $\ddot{x}(t)+(\alpha /t+\gamma )\dot{x}(t)+\nabla g(x(t))=0$ for almost every $t\geq t_{0}$;
3. $x(t_{0})=u_{0}$ and $\dot{x}(t_{0})=v_{0}$.

Recall that a function $x:[t_{0},+\infty )\to R^{n}$ is absolutely continuous on an interval $\left[t_{0},T\right]$, if there exists an integrable function $y:[t_{0},T]\to R^{n}$ such that

$$x(t)=x(0)+\int_{t_{0}}^{t}y(s)\;ds\;\forall \;t\in [t_{0},T].$$

It follows from the definition that an absolutely continuous function is differentiable almost everywhere, its derivative coincides with its distributional derivative almost everywhere and one can recover the function from its derivative $\dot{x}=y$ by the integration formula above. On the other hand, if $x:[t_{0},T]\to R^{n}$ (where $T>t_{0}$) is absolutely continuous and $B:R^{n}\to R^{n}$ is *L*-Lipschitz continuous (where L≥0), then the function B∘x is absolutely continuous, too. Moreover, B∘x is almost everywhere differentiable and the inequality $∥(d/dt)B(x(t))∥\leq L∥\dot{x}(t)∥$ holds for almost every $t\geq t_{0}$ [see [14,15]].

We prove existence and uniqueness of a strong global solution of (2) by making use of the Cauchy–Lipschitz–Picard Theorem for absolutely continues trajectories [see for example [16, Proposition 6.2.1], [17, Theorem 54]]. The key argument is that one can rewrite (2) as a particular first-order dynamical system in a suitably chosen product space [see also [18]].

Theorem 2.1 For every starting points $u_{0},v_{0}\in R^{n}$ there exists a unique strong global solution of the dynamical system (2).

Proof. By making use of the notation $X(t)=(x(t),\dot{x}(t))$, the system (2) can be rewritten as a first-order dynamical system:

(6) $$\begin{array}{r} \begin{array}{rl} \dot{X}(t) & =F(t,X(t)),\\ X(t_{0}) & =(u_{0},v_{0}), \end{array} \end{array}$$

where $F:[t_{0},+\infty )\times R^{n}\times R^{n}⟶R^{n}\times R^{n},\;F(t,u,v)=(v,-(\alpha /t+\gamma )v-\nabla g(u)).$

First we show that F(t,⋅,⋅) is L(t)-Lipschitz continuous for every $t\geq t_{0}$ and that the Lipschitz constant is a function of time with the property that $L(\cdot )\in L_{\mathrm{loc}}^{1}([t_{0},+\infty ))$. Indeed, for every $(u,v),(\bar{u},\bar{v})\in R^{n}\times R^{n}$, we have

$$\begin{array}{rl} ∥F(t,u,v)-F(t,\bar{u},\bar{v})∥ & =\sqrt{∥v-\bar{v}{∥}^{2}+{∥\left(\frac{\alpha }{t}+\gamma \right)(\bar{v}-v)+(\nabla g(\bar{u})-\nabla g(u))∥}^{2}}\\ & \leq \sqrt{\left(1+2{\left(\frac{\alpha }{t}+\gamma \right)}^{2}\right)∥v-\bar{v}{∥}^{2}+2L_{g}^{2}∥u-\bar{u}{∥}^{2}}\\ & \leq \sqrt{1+2L_{g}^{2}+2{\left(\frac{\alpha }{t}+\gamma \right)}^{2}}\sqrt{∥v-\bar{v}{∥}^{2}+∥u-\bar{u}{∥}^{2}}\\ & =\sqrt{1+2L_{g}^{2}+2{\left(\frac{\alpha }{t}+\gamma \right)}^{2}}∥(u,v)-(\bar{u},\bar{v})∥. \end{array}$$

Obviously, the Lipschitz constant function $t\mapsto L(t):=\sqrt{1+2L_{g}^{2}+2(\alpha /t+\gamma {)}^{2}}$ is continuous, hence integrable, on $\left[t_{0},T\right]$ for all $t_{0}<T<+\infty$, consequently, $L\in L_{\mathrm{loc}}^{1}([t_{0},+\infty ))$.

Next we show that $F(\cdot ,u,v)\in L_{\mathrm{loc}}^{1}([t_{0},+\infty ),R^{n}\times R^{n})$ for all $u,v\in R^{n}$. Let $u,v\in R^{n}$ be fixed. For $t_{0}<T<+\infty$, one has

$$\begin{array}{rl} \int_{t_{0}}^{T}∥F(t,u,v)∥\;dt & =\int_{t_{0}}^{T}\sqrt{∥v{∥}^{2}+{∥\left(\frac{\alpha }{t}+\gamma \right)v+\nabla g(u)∥}^{2}}dt\\ & \leq \int_{t_{0}}^{T}\sqrt{\left(1+2{\left(\frac{\alpha }{t}+\gamma \right)}^{2}\right)∥v{∥}^{2}+2∥\nabla g(u){∥}^{2}}\;dt\\ & \leq \sqrt{∥v{∥}^{2}+∥\nabla g(u){∥}^{2}}\int_{t_{0}}^{T}\sqrt{3+2{\left(\frac{\alpha }{t}+\gamma \right)}^{2}}dt \end{array}$$

and the conclusion follows by the continuity of $t\mapsto \sqrt{3+2(\alpha /t+\gamma {)}^{2}}$ on $\left[t_{0},T\right]$.

The Cauchy–Lipschitz–Picard Theorem guarantees existence and uniqueness of the trajectory of the first-order dynamical system (6) and thus of the second-order dynamical system (2).

The next result shows that the acceleration of the trajectory generated by (2) is also locally absolutely continuous on $\left[t_{0},+\infty \right)$.

Proposition 2.1 For the starting points $u_{0},v_{0}\in R^{n},$ let *x* be the unique strong global solution of (2). Then $\ddot{x}$ is locally absolutely continuous on $[t_{0},+\infty ),$ hence the third-order derivative $x^{\left(3\right)}$ exists almost everywhere on $[t_{0},+\infty ).$

Proof. Let *T*>0 be fixed. According to Theorem 2.1, $X(t):=(x(t),\dot{x}(t))$ is absolutely continuous on $\left[t_{0},T\right]$. We endow the product space $R^{n}\times R^{n}$ with the 1-norm. For arbitrary $s,t\in [t_{0},T]$, we have

$$\begin{array}{rl} ∥\dot{X}(s)-\dot{X}(t){∥}_{1} & =∥F(s,X(s))-F(t,X(t)){∥}_{1}\\ & ={∥\left(\dot{x}(s)-\dot{x}(t),-\left(\frac{\alpha }{s}+\gamma \right)\dot{x}(s)+\left(\frac{\alpha }{t}+\gamma \right)\dot{x}(t)-\nabla g(x(s))+\nabla g(x(t))\right)∥}_{1}\\ & \leq (1+\gamma )∥\dot{x}(s)-\dot{x}(t)∥+∥\frac{\alpha }{s}\dot{x}(s)-\frac{\alpha }{t}\dot{x}(t)∥+∥\nabla g(x(s))-\nabla g(x(t))∥\\ & \leq (1+\gamma )∥\dot{x}(s)-\dot{x}(t)∥+\frac{|\alpha |}{s}∥\dot{x}(s)-\dot{x}(t)∥+∥\frac{\alpha }{s}\dot{x}(t)-\frac{\alpha }{t}\dot{x}(t)∥\;+L_{g}∥x(s)-x(t)∥\\ & \leq L_{1}∥\dot{x}(s)-\dot{x}(t)∥+L_{2}\left|\frac{\alpha }{s}-\frac{\alpha }{t}\right|+L_{g}∥x(s)-x(t)∥, \end{array}$$

where

$$L_{1}:=\max_{t\in [t_{0},T]}\left(1+\gamma +\frac{|\alpha |}{t}\right)\;\text{and}\;L_{2}:=\max_{t\in [t_{0},T]}∥\dot{x}(t)∥.$$

Let be ε>0. Since the functions $\dot{x}(\cdot ),t\mapsto \alpha /t$ and x(⋅) are absolutely continuous on $\left[t_{0},T\right]$, there exists η>0 such that for any finite family of intervals $I_{k}=(a_{k},b_{k})\subseteq [t_{0},T]$, the implication

$$\begin{array}{rl} & \left(I_{k}\cap I_{j}=\emptyset \text{ and }\sum_{k}|b_{k}-a_{k}|<\eta \right)\\ & \;⟹\sum_{k}∥\dot{x}(b_{k})-\dot{x}(a_{k})∥<\frac{\varepsilon }{3L_{1}},\;\sum_{k}\left|\frac{\alpha }{b_{k}}-\frac{\alpha }{a_{k}}\right|<\frac{\varepsilon }{3L_{2}}\;\text{and}\;\sum_{k}∥x(b_{k})-x(a_{k})∥<\frac{\varepsilon }{3L_{g}} \end{array}$$

holds. Consequently,

$$\sum_{k}∥\dot{X}(a_{k})-\dot{X}(b_{k})∥<\frac{\varepsilon }{3}+\frac{\varepsilon }{3}+\frac{\varepsilon }{3}=\varepsilon ,$$

hence $\dot{X}(\cdot )=(\dot{x}(\cdot ),\ddot{x}(\cdot ))$ is absolutely continuous on $\left[t_{0},T\right]$, which shows that $\ddot{x}$ is absolutely continuous $\left[t_{0},T\right]$. This proves that $\ddot{x}$ is locally absolutely continuous on $\left[t_{0},+\infty \right)$, which means that the third-order derivative $x^{\left(3\right)}$ exists almost everywhere on $[t_{0},+\infty ).$

The following results provides an estimate for the third-order derivative of the strong global solution of the dynamical system (2) in terms its first- and second-order derivatives.

Lemma 2.1 For the starting points $u_{0},v_{0}\in R^{n},$ let *x* be the unique strong global solution of (2). Then, for almost every $t\in [t_{0},+\infty ),$ it holds

(7) $$∥x^{\left(3\right)}(t)∥\leq \left(L_{g}+\frac{|\alpha |}{t^{2}}\right)∥\dot{x}(t)∥+\left(\gamma +\frac{|\alpha |}{t}\right)∥\ddot{x}(t)∥.$$

Proof. Let $t\in [t_{0},+\infty )$ be such that $\dot{X}(t)=F(t,X(t))$. We have for almost every *h*>0 that

$$\begin{array}{rl} & ∥\dot{X}(t+h)-\dot{X}(t){∥}_{1}=∥F(t+h,X(t+h))-F(t,X(t)){∥}_{1}\\ & \;={∥\left(\dot{x}(t+h)-\dot{x}(t),-\left(\frac{\alpha }{t+h}+\gamma \right)\dot{x}(t+h)+\left(\frac{\alpha }{t}+\gamma \right)\dot{x}(t)-\nabla g(x(t+h))+\nabla g(x(t))\right)∥}_{1}\\ & \;=∥\dot{x}(t+h)-\dot{x}(t)∥+∥-\left(\frac{\alpha }{t+h}+\gamma \right)\dot{x}(t+h)+\left(\frac{\alpha }{t}+\gamma \right)\dot{x}(t)-\nabla g(x(t+h))+\nabla g(x(t))∥\\ & \;\leq (1+\gamma )∥\dot{x}(t+h)-\dot{x}(t)∥+∥\frac{\alpha }{t+h}\dot{x}(t+h)-\frac{\alpha }{t}\dot{x}(t)∥+∥\nabla g(x(t+h))-\nabla g(x(t))∥\\ & \;\leq (1+\gamma )∥\dot{x}(t+h)-\dot{x}(t)∥+∥\frac{\alpha }{t+h}\dot{x}(t+h)-\frac{\alpha }{t}\dot{x}(t)∥+L_{g}∥x(t+h)-x(t)∥. \end{array}$$

Hence,

$$\begin{array}{rl} {∥\frac{\dot{X}(t+h)-\dot{X}(t)}{h}∥}_{1} & \leq (1+\gamma )∥\frac{\dot{x}(t+h)-\dot{x}(t)}{h}∥+∥\frac{\left(\alpha /(t+h))\dot{x}(t+h)-(\alpha /t)\dot{x}(t\right)}{h}∥\\ & \;+L_{g}∥\frac{x(t+h)-x(t)}{h}∥. \end{array}$$

By taking the limit as h→0, we obtain

$$∥\ddot{X}(t){∥}_{1}\leq (1+\gamma )∥\ddot{x}(t)∥+∥{\left(\frac{\alpha }{t}\dot{x}(t)\right)}^{\prime }∥+L_{g}∥\dot{x}(t)∥.$$

Since $∥\ddot{X}(t){∥}_{1}=∥x^{\left(3\right)}(t)∥+∥\ddot{x}(t)∥$, we conclude

$$∥x^{\left(3\right)}(t)∥\leq \left(L_{g}+\frac{|\alpha |}{t^{2}}\right)∥\dot{x}(t)∥+\left(\gamma +\frac{|\alpha |}{t}\right)∥\ddot{x}(t)∥.$$

Remark 2.1 For

$$N:=\max_{t\geq t_{0}}\left(L_{g}+\frac{|\alpha |}{t^{2}},\gamma +\frac{|\alpha |}{t}\right),$$

we have that

$$∥x^{\left(3\right)}(t)∥\leq N(∥\ddot{x}(t)∥+∥\dot{x}(t)∥)$$

for almost every $t\in [t_{0},+\infty )$.

## 3. Convergence of trajectories

In this section, we study the convergence of the trajectory of the dynamical system (2). We denote by

$$\omega (x):=\{\bar{x}\in R^{n}:\exists t_{k}⟶+\infty \text{ such that }x(t_{k})⟶\bar{x}\;\text{as}\;k⟶+\infty \}$$

the set of limit points of the trajectory *x*.

Before proving a first result in this sense, we recall two technical lemmas which will play an essential role in the asymptotic analysis.

Lemma 3.1 Suppose that F:[0,+∞)→R is locally absolutely continuous and bounded below and that there exists $G\in L^{1}([0,+\infty ))$ such that for almost every t∈[0,+∞)

$$\frac{d}{dt}F(t)\leq G(t).$$

Then there exists $\lim_{t\to +\infty }F(t)\in R$.

Lemma 3.2 Suppose that F:[0,+∞)→[0,+∞) is locally absolutely continuous and $F\in L^{p}([0,+\infty )),1\leq p<\infty ,$ and that there exists $G:[0,+\infty )\to R,G\in L^{r}([0,+\infty )),1\leq r\leq \infty ,$ such that for almost every t∈[0,+∞)

$$\frac{d}{dt}F(t)\leq G(t).$$

Then it holds $\lim_{t\to +\infty }F(t)=0$.

Theorem 3.1 Assume that *g* is bounded from below and, for $u_{0},v_{0}\in R^{n},$ let *x* be the unique strong global solution of the dynamical system (2). Then the following statements are true:

1. $\dot{x},\;\ddot{x}\in L^{2}([t_{0},+\infty ),R^{n});$
2. there exists β>0 such that the limit limt⟶+∞g(βa˙x(t)+x(t)) exists and is finite;
3. $\lim_{t⟶+\infty }\ddot{x}(t)=0$ and $\lim_{t⟶+\infty }\dot{x}(t)=0;$
4. ω(x)⊆crit(g).

Proof. Choose $0<\beta <\min(2/L_{g},(\sqrt{L_{g}^{2}+2\gamma L_{g}}-L_{g})/L_{g})$. By using the $L_{g}$-Lipschitz continuity of ∇g, for almost every $t\in [t_{0},+\infty )$ it holds

$$\begin{array}{rl} \frac{d}{dt}g(\beta \dot{x}(t)+x(t)) & =\langle \beta \ddot{x}(t)+\dot{x}(t),\nabla g(\beta \dot{x}(t)+x(t))\rangle \\ & =\langle \beta \ddot{x}(t)+\dot{x}(t),\nabla g(\beta \dot{x}(t)+x(t))-\nabla g(x(t))\rangle \\ & \;+\left\langle \beta \ddot{x}(t)+\dot{x}(t),-\ddot{x}(t)-\left(\frac{\alpha }{t}+\gamma \right)\dot{x}(t)\right\rangle \\ & \leq -\beta ∥\ddot{x}(t){∥}^{2}-\left(1+\beta \gamma +\frac{\alpha \beta }{t}\right)\langle \ddot{x}(t),\dot{x}(t)\rangle -\left(\gamma +\frac{\alpha }{t}\right)∥\dot{x}(t){∥}^{2}\\ & \;+L_{g}∥\beta \ddot{x}(t)+\dot{x}(t)∥∥\beta \dot{x}(t)∥. \end{array}$$

Taking into account that

$$∥\beta \ddot{x}(t)+\dot{x}(t)∥∥\beta \dot{x}(t)∥\leq \beta^{2}∥\ddot{x}(t)∥∥\dot{x}(t)∥+\beta ∥\dot{x}(t){∥}^{2}\leq \frac{1}{2}\beta^{2}∥\ddot{x}(t){∥}^{2}+\left(\beta +\frac{1}{2}\beta^{2}\right)∥\dot{x}(t){∥}^{2}$$

and

$$-\left(1+\beta \gamma +\frac{\alpha \beta }{t}\right)\langle \ddot{x}(t),\dot{x}(t)\rangle =-\frac{1}{2}\frac{d}{dt}\left[\left(1+\beta \gamma +\frac{\alpha \beta }{t}\right)∥\dot{x}(t){∥}^{2}\right]-\frac{\alpha \beta }{2t^{2}}∥\dot{x}(t){∥}^{2},$$

we obtain for almost every $t\in [t_{0},+\infty )$

(8) $$\begin{array}{rl} & \frac{d}{dt}\left(g(\beta \dot{x}(t)+x(t))+\frac{1}{2}\left(1+\beta \gamma +\frac{\alpha \beta }{t}\right)∥\dot{x}(t){∥}^{2}\right)\\ & \;\leq \left(-\beta +\frac{L_{g}\beta^{2}}{2}\right)∥\ddot{x}(t){∥}^{2}+\left(-\gamma +L_{g}\beta +\frac{L_{g}\beta^{2}}{2}-\frac{\alpha }{t}-\frac{\alpha \beta }{2t^{2}}\right)∥\dot{x}(t){∥}^{2}. \end{array}$$

Since $0<\beta <\min(2/L_{g},(\sqrt{L_{g}^{2}+2\gamma L_{g}}-L_{g})/L_{g})$, there exists $t_{1}>0$ such that for every $t\geq t_{1}$ it holds

(9) $$1+\beta \gamma +\frac{\alpha \beta }{t}>0,\;-\beta +\frac{L_{g}\beta^{2}}{2}<0\;\text{and}\;-\gamma +L_{g}\beta +\frac{L_{g}\beta^{2}}{2}-\frac{\alpha }{t}-\frac{\alpha \beta }{2t^{2}}<0.$$

For simplicity, we denote

$$A:=-\beta +\frac{L_{g}\beta^{2}}{2}\;\text{and}\;B(t):=-\gamma +L_{g}\beta +\frac{L_{g}\beta^{2}}{2}-\frac{\alpha }{t}-\frac{\alpha \beta }{2t^{2}}\;\forall \;t\in [t_{0},+\infty ).$$

Let be $T>t_{1}.$ Since $x\in C^{2}([t_{1},T],R^{n})$, we have $x,\dot{x},\ddot{x}\in L^{2}([t_{1},T],R^{n}).$ Furthermore, by the $L_{g}$-Lipschitz property of ∇g, it holds $\nabla g\in L^{2}([t_{1},T],R^{n}).$ By integrating (8) on $\left[t_{1},T\right]$, we obtain

(10) $$\begin{array}{rlr} & g(\beta \dot{x}(T)+x(T))+\frac{1}{2}\left(1+\beta \gamma +\frac{\alpha \beta }{T}\right)∥\dot{x}(T){∥}^{2}-\int_{t_{1}}^{T}A∥\ddot{x}(t){∥}^{2}dt-\int_{t_{1}}^{T}B(t)∥\dot{x}(t){∥}^{2}dt\\ & \;\leq g(\beta \dot{x}(t_{1})+x(t_{1}))+\frac{1}{2}\left(1+\beta \gamma +\frac{\alpha \beta }{t_{1}}\right)∥\dot{x}(t_{1}){∥}^{2}. & \end{array}$$

Taking into account that *g* is bounded from bellow, by letting T⟶+∞, we obtain

$$\int_{t_{1}}^{\infty }(-A∥\ddot{x}(t){∥}^{2})\;dt<+\infty \;\text{and}\;\int_{t_{1}}^{\infty }(-B(t)∥\dot{x}(t){∥}^{2})\;dt<+\infty$$

Consequently $∥\ddot{x}(\cdot ){∥}^{2},\;B(\cdot )∥\dot{x}(\cdot ){∥}^{2}\in L^{1}([t_{0},+\infty ),R)$, hence

$$\ddot{x},\;\dot{x}\in L^{2}([t_{0},+\infty ),R^{n}).$$

On the other hand, (8) and Lemma 3.1 ensure that the limit

(11) $$\lim_{t⟶+\infty }\left(g(\beta \dot{x}(t)+x(t))+\frac{1}{2}\left(1+\beta \gamma +\frac{\alpha \beta }{t}\right)∥\dot{x}(t){∥}^{2}\right)$$

exists and is finite.

As for almost every $t\in [t_{0},+\infty )$

$$\frac{d}{dt}(∥\dot{x}(t){∥}^{2})=2\langle \ddot{x}(t),\dot{x}(t)\rangle \leq ∥\dot{x}(t){∥}^{2}+∥\ddot{x}(t){∥}^{2}$$

and $∥\dot{x}(\cdot ){∥}^{2}+∥\ddot{x}(\cdot ){∥}^{2}\in L^{1}([t_{0},+\infty ))$, according to Lemma 3.2, it follows that $\lim_{t⟶+\infty }\dot{x}(t)=0.$

As for almost every $t\in [t_{0},+\infty )$

$$\frac{d}{dt}(∥\ddot{x}(t){∥}^{2})=2\langle x^{\left(3\right)}(t),\ddot{x}(t)\rangle \leq ∥x^{\left(3\right)}(t){∥}^{2}+∥\ddot{x}(t){∥}^{2}$$

and $∥x^{\left(3\right)}(\cdot ){∥}^{2}+∥\ddot{x}(\cdot ){∥}^{2}\in L^{1}([t_{0},+\infty ))$ (see Remark 2.1 and (i)), according to Lemma 3.2, it follows that $\lim_{t⟶+\infty }\ddot{x}(t)=0.$

Finally, by using that $\lim_{t⟶+\infty }\dot{x}(t)=0$, (11) becomes

(12) $$\exists \lim_{t⟶+\infty }\left(g(\beta \dot{x}(t)+x(t))+\frac{1}{2}\left(1+\beta \gamma +\frac{\alpha \beta }{t}\right)∥\dot{x}(t){∥}^{2}\right)=\lim_{t⟶+\infty }g(\beta \dot{x}(t)+x(t))\in R.$$

Let $\bar{x}\in \omega (x).$ Then there exists a sequence $t_{k}⟶+\infty ,\;k⟶+\infty$ such that $x(t_{k})⟶\bar{x}$ as k⟶+∞. By using the continuity of ∇g, we have

$$0=\lim_{k⟶+\infty }\left(\ddot{x}(t_{k})+\left(\frac{\alpha }{t_{k}}+\gamma \right)\dot{x}(t_{k})+\nabla g(x(t_{k}))\right)=\nabla g(\bar{x}),$$

which shows that $\bar{x}\in \mathrm{crit}(g).$

In the following result, we use the distance function to a set, defined for $A\subseteq R^{n}$ as $\mathrm{dist}(x,A)=\underset{y\in A}{\inf}∥x-y∥$ for all $x\in R^{n}$. The following result is a direct consequence of Theorem 3.1.

Lemma 3.3 Assume that *g* is bounded from below and, for $u_{0},v_{0}\in R^{n},$ let *x* be the unique strong global solution of the dynamical system (2). Define

$$H:R^{n}\times R^{n}⟶R,\;H(x,y)=g(x)+\frac{1}{2}∥x-y{∥}^{2}.$$

Let be $0<\beta <\min(2/L_{g},(\sqrt{L_{g}^{2}+2\gamma L_{g}}-L_{g})/L_{g})$ and $t_{1}>0$ such that for every $t\geq t_{1}$ the inequalities (9) hold. For every $t\in [t_{1},+\infty ),$ define

$$\begin{array}{rl} u(t) & :=\beta \dot{x}(t)+x(t),\;v(t):=\left(\sqrt{1+\beta \gamma +\frac{\alpha \beta }{t}}+\beta \right)\dot{x}(t)+x(t),\\ A & =-\beta +\frac{L_{g}\beta^{2}}{2},\;B(t):=-\gamma +L_{g}\beta +\frac{L_{g}\beta^{2}}{2}-\frac{\alpha }{t}-\frac{\alpha \beta }{2t^{2}}\;and\\ p(t) & :=L_{g}\beta +\gamma +\frac{|\alpha |}{t}+2\sqrt{1+\beta \gamma +\frac{\alpha \beta }{t}}. \end{array}$$

Then the following statements are true:

1. ω(u)=ω(v)=ω(x);
2. $\frac{d}{dt}H(u(t),v(t))\leq A∥\ddot{x}(t){∥}^{2}+B(t)∥\dot{x}(t){∥}^{2}\leq 0\text{ for almost every }t\geq t_{1};$
3. the limit limt⟶+∞H(u(t),v(t))=limt⟶+∞g(βa˙x(t)+x(t)) exists and is finite;
4. *H* is finite and constant on $\omega (u,v)=\{(\bar{x},\bar{x})\in R^{n}\times R^{n}:\bar{x}\in \omega (x)\};$
5. $∥\nabla H(u(t),v(t))∥\leq ∥\ddot{x}(t)∥+p(t)∥\dot{x}(t)∥\text{ for almost every }t\geq t_{1};$
6. ω(u,v)⊆crit(H).

If *x* is bounded, then

1. ω(u,v) is nonempty and compact;
2. $\lim_{t⟶+\infty }\mathrm{dist}((u(t),v(t)),\omega (u,v))=0.$

Proof. (i) According to Theorem 3.1(iii),

$$\lim_{t⟶+\infty }\beta \dot{x}(t)=\lim_{t⟶+\infty }\left(\sqrt{1+\beta \gamma +\frac{\alpha \beta }{t}}+\beta \right)\dot{x}(t)=0,$$

hence ω(u)=ω(v)=ω(x).

(ii) and (iii) are nothing else than (8) and (12), respectively.

(iv) follows directly from (iii).

(v) Since ∇H(x,y)=(∇g(x)+x−y,y−x) for every $(x,y)\in R^{n}\times R^{n}$, by using (2), we have for almost every $t\in [t_{1},+\infty )$

$$\begin{array}{rl} & ∥\nabla H(u(t),v(t))∥\leq ∥\nabla g(u(t))∥+2∥u(t)-v(t)∥\\ & \;\leq ∥\nabla g(u(t))-\nabla g(x(t))∥+∥\nabla g(x(t))∥+2∥u(t)-v(t)∥\\ & \;\leq L_{g}\beta ∥\dot{x}(t)∥+∥-\ddot{x}(t)-\left(\gamma +\frac{\alpha }{t}\right)\dot{x}(t)∥+2∥u(t)-v(t)∥\\ & \;\leq ∥\ddot{x}(t)∥+\left(L_{g}\beta +\gamma +\frac{|\alpha |}{t}+2\sqrt{1+\beta \gamma +\frac{\alpha \beta }{t}}\right)∥\dot{x}(t)∥=∥\ddot{x}(t)∥+p(t)∥\dot{x}(t)∥. \end{array}$$

(vi) Since

$$\mathrm{crit}H=\{(x,y)\in R^{n}\times R^{n}:\nabla H(x,y)=(0,0)\}=\{(\bar{x},\bar{x})\in R^{n}\times R^{n}:\bar{x}\in \mathrm{crit}(g)\}$$

and (see (i))

$$\omega (u,v)=\{(\bar{x},\bar{x})\in R^{n}\times R^{n}:\bar{x}\in \omega (x)\},$$

by Theorem 3.1(iv) one obtains

ω(u,v)⊆crit(H).

Assume that *x* is bounded.

(vii) Since $\dot{x}(t)⟶0,\;t⟶+\infty ,$ we obtain that *u* and *v* are bounded, too. Thus, the set of limit points ω(u,v) is nonempty. Furthermore, since $\omega (u,v)=\{(\bar{x},\bar{x})\in R^{n}\times R^{n}:\bar{x}\in \omega (x)\}$ and ω(x) is bounded, it is enough to show that ω(x) is closed.

Let be $\left({\bar{x}}_{n}{)}_{n\geq 1}\subseteq \omega (x\right)$ and assume that $\lim_{n⟶+\infty }{\bar{x}}_{n}=x^{*}.$ We show that $x^{*}\in \omega (x).$ Obviously, for every n≥1, there exists a sequence $t_{k}^{n}⟶+\infty ,\;k⟶+\infty$, such that

$$\lim_{k⟶+\infty }x(t_{k}^{n})={\bar{x}}_{n}.$$

Let be ϵ>0. Since $\lim_{n⟶+\infty }{\bar{x}}_{n}=x^{*},$ there exists N(ϵ)∈N such that for every n≥N(ϵ) it holds

$$∥{\bar{x}}_{n}-x^{*}∥<\frac{\epsilon }{2}.$$

Let n≥1 be fixed. Since $\lim_{k⟶+\infty }x(t_{k}^{n})={\bar{x}}_{n},$ there exists k(n,ϵ)∈N such that for every k≥k(n,ϵ) it holds

$$∥x(t_{k}^{n})-{\bar{x}}_{n}∥<\frac{\epsilon }{2}.$$

Let be $k_{n}\geq k(n,\varepsilon )$ such that $t_{k_{n}}^{n}>n$. Obviously $t_{k_{n}}^{n}⟶\infty$ as n⟶+∞ and for every n≥N(ϵ)

$$∥x(t_{k_{n}}^{n})-x^{*}∥<\epsilon .$$

Hence,

$$\lim_{n⟶+\infty }x(t_{k_{n}}^{n})=x^{*},$$

thus $x^{*}\in \omega (x).$

(viii) follows from the definition of the set ω(u,v).

Remark 3.1 Combining (iii) and (iv) in Lemma 3.3, it follows that for every $\bar{x}\in \omega (x)$ and $t_{k}⟶+\infty$ such that $x(t_{k})⟶\bar{x}$ as k⟶+∞ we have

$$\lim_{k⟶+\infty }H(u(t_{k}),v(t_{k}))=H(\bar{x},\bar{x}).$$

The convergence of the trajectory generated by the dynamical system (2) will be shown in the framework of functions satisfying the *Kurdyka–Łojasiewicz property*. For η∈(0,+∞], we denote by $\Theta_{\eta }$ the class of concave and continuous functions ϕ:[0,η)→[0,+∞) such that ϕ(0)=0, *ϕ* is continuously differentiable on (0,η), continuous at 0 and $\phi^{\prime }(s)>0$ for all s∈(0,η).

Definition 3.1 **Kurdyka–Łojasiewicz property**

Let $f:R^{n}\to R$ be a differentiable function. We say that *f* satisfies the *Kurdyka–Łojasiewicz (KL) property* at $\bar{x}\in R^{n}$ if there exist η∈(0,+∞], a neighbourhood *U* of $\bar{x}$ and a function $\phi \in \Theta_{\eta }$ such that for all *x* in the intersection

$$U\cap \{x\in R^{n}:f(\bar{x})<f(x)<f(\bar{x})+\eta \},$$

the following inequality holds

$$\phi^{\prime }(f(x)-f(\bar{x}))∥\nabla f(x))∥\geq 1.$$

If *f* satisfies the KL property at each point in $R^{n}$, then *f* is called a *KL function*.

The origins of this notion go back to the pioneering work of Łojasiewicz [19], where it is proved that for a real-analytic function $f:R^{n}\to R$ and a critical point $\bar{x}\in R^{n}$ (that is $\nabla f(\bar{x})=0$), there exists θ∈[1/2,1) such that the function $|f-f(\bar{x}){|}^{\theta }∥\nabla f{∥}^{-1}$ is bounded around $\bar{x}$. This corresponds to the situation when $\phi (s)=C(1-\theta {)}^{-1}s^{1-\theta }$. The result of Łojasiewicz allows the interpretation of the KL property as a re-parametrization of the function values in order to avoid flatness around the critical points. Kurdyka [20] extended this property to differentiable functions definable in an o-minimal structure. Further extensions to the nonsmooth setting can be found in [21–24].

To the class of KL functions belong semi-algebraic, real sub-analytic, semiconvex, uniformly convex and convex functions satisfying a growth condition. We refer the reader to [21–27] and the references therein for more details regarding all the classes mentioned above and illustrating examples.

An important role in our convergence analysis will be played by the following uniformized KL property given in [27, Lemma 6].

Lemma 3.4 Let $\Omega \subseteq R^{n}$ be a compact set and let $f:R^{n}\to R$ be a differentiable function. Assume that *f* is constant on Ω and *f* satisfies the KL property at each point of Ω. Then there exist ϵ,η>0 and $\phi \in \Theta_{\eta }$ such that for all $\bar{x}\in \Omega$ and for all *x* in the intersection

(13) $$\{x\in R^{n}:\mathrm{dist}(x,\Omega )<\varepsilon \}\cap \{x\in R^{n}:f(\bar{x})<f(x)<f(\bar{x})+\eta \},$$

the following inequality holds

(14) $$\phi^{\prime }(f(x)-f(\bar{x}))∥\nabla f(x)∥\geq 1.$$

The following theorem is the main result of the paper and concerns the global asymptotic convergence of the trajectory generated by (2).

Theorem 3.2 Assume that *g* is bounded from below and, for $u_{0},v_{0}\in R^{n},$ let *x* be the unique strong global solution of (2). Suppose that

$$H:R^{n}\times R^{n}⟶R,\;H(x,y)=g(x)+\frac{1}{2}∥x-y{∥}^{2}$$

is a KL function and *x* is bounded. Then the following statements are true:

1. $\dot{x},\;\ddot{x}\in L^{1}([t_{0},+\infty ),R^{n});$
2. there exists $\bar{x}\in \mathrm{crit}(g)$ such that $\lim_{t⟶+\infty }x(t)=\bar{x}.$

Proof. Let be $0<\beta <\min(2/L_{g},(\sqrt{L_{g}^{2}+2\gamma L_{g}}-L_{g})/L_{g})$ and $t_{1}>0$ such that for every $t\geq t_{1}$ the inequalities (9) hold. Furthermore, we will use the notations made in Lemma 3.3, according to which we can choose $\left(\tilde{x},\tilde{x})\in \omega (u,v\right)$. It holds

$$\lim_{t⟶+\infty }H(u(t),v(t))=H(\tilde{x},\tilde{x}).$$

*Case I*. There exists $\bar{t}\geq t_{1}$ such that $H(u(\bar{t}),v(\bar{t}))=H(\tilde{x},\tilde{x}).$ From Lemma 3.3(ii), we have

$$\frac{d}{dt}H(u(t),v(t))\leq A∥\ddot{x}(t){∥}^{2}+B(t)∥\dot{x}(t){∥}^{2}\leq 0\text{ for almost every }t\geq t_{1},$$

hence

$$H(u(t),v(t))\leq H(\tilde{x},\tilde{x})\text{ for every }t\geq \bar{t}.$$

On the other hand,

$$H(u(t),v(t))\geq \lim_{t⟶+\infty }H(u(t),v(t))=H(\tilde{x},\tilde{x})\;\text{ for every }t\geq t_{1},$$

hence

$$H(u(t),v(t))=H(\tilde{x},\tilde{x})\text{ for every }t\geq \bar{t}.$$

Hence, (d/dt)H(u(t),v(t))=0, which leads to

$$0\leq A∥\ddot{x}(t){∥}^{2}+B(t)∥\dot{x}(t){∥}^{2}\leq 0\text{ for almost every }t\geq \bar{t}.$$

Since *A*<0 and B(t)<0 for every $t\geq t_{1}$, the latter inequality can hold only if

$$\dot{x}(t)=\ddot{x}(t)=0\text{ for almost every }t\geq \bar{t}.$$

Consequently, $\dot{x},\ddot{x}\in L^{1}([t_{0},+\infty ),R^{n})$ and *x* is constant on $[\bar{t},+\infty ).$

*Case II*. We assume that $H(u(t),v(t))>H(\tilde{x},\tilde{x})$ holds for every $t\geq t_{1}$. Let Ω:=ω(u,v). According to Lemma 3.3, Ω is nonempty and compact and *H* is constant on Ω. Since *H* is a KL function, according to Lemma 3.4, there exist ε,η>0 and $\phi \in \Theta_{\eta }$ such that for every $\left(\tilde{z},\tilde{w}\right)$ in the intersection

$$\left\{(z,w)\in R^{n}\times R^{n}:\mathrm{dist}((z,w),\Omega )<\varepsilon \}\cap \{(z,w)\in R^{n}\times R^{n}:H(\bar{x},\bar{x})<H(z,w)<H(\tilde{x},\tilde{x})+\eta \right\}$$

one has

$$\phi^{\prime }(H(\tilde{z},\tilde{w})-H(\tilde{x},\tilde{x}))∥\nabla H(\tilde{z},\tilde{w})∥\geq 1.$$

Since $\lim_{t⟶+\infty }\mathrm{dist}(u(t),v(t),\Omega )=0$, there exists $t_{2}\geq t_{1}$ such that dist(u(t),v(t)),Ω)<ϵ for every $t\geq t_{2}$. Since $\lim_{t⟶+\infty }H(u(t),v(t))=H(\bar{x},\bar{x})$, there exists $t_{3}\geq t_{1}$ such that $H(\bar{x},\bar{x})<H(u(t),v(t))<H(\bar{x},\bar{x})+\eta$ for every $t\geq t_{3}$. Hence, for every $t\geq T:=\max(t_{2},t_{3})$, we have

$$\phi^{\prime }(H(u(t),v(t))-H(\bar{x},\bar{x}))\cdot ∥\nabla H(u(t),v(t))∥\geq 1.$$

According to Lemma 3.3 (ii) and (v), we have $(d/dt)H(u(t),v(t))\leq A∥\ddot{x}(t){∥}^{2}+B(t)∥\dot{x}(t){∥}^{2}\leq 0$ and $∥\nabla H(u(t),v(t))∥\leq ∥\ddot{x}(t)∥+p(t)∥\dot{x}(t)∥,$ hence

$$\begin{array}{rl} \frac{d}{dt}\phi (H(u(t),v(t))-H(\tilde{x},\tilde{x})) & =\phi^{\prime }(H(u(t),v(t))-H(\tilde{x},\tilde{x}))\frac{d}{dt}H(u(t),v(t))\\ & \leq \frac{A∥\ddot{x}(t){∥}^{2}+B(t)∥\dot{x}(t){∥}^{2}}{∥\ddot{x}(t)∥+p(t)∥\dot{x}(t)∥} \end{array}$$

for almost every t∈[T,+∞).

By integrating on the interval $\left[T,\bar{T}\right]$, for $\bar{T}>T$, we obtain

$$\phi (H(u(\bar{T}),v(\bar{T}))-H(\tilde{x},\tilde{x}))-\int_{T}^{\bar{T}}\frac{A∥\ddot{x}(s){∥}^{2}+B(s)∥\dot{x}(s){∥}^{2}}{∥\ddot{x}(s)∥+p(s)∥\dot{x}(s)∥}\;ds\leq \phi (H(u(T),v(T))-H(\tilde{x},\tilde{x})).$$

Since *ϕ* is bounded from below, A<0,B(s)<0 and p(s)>0 for every s≥T and $\bar{T}$ was arbitrarily chosen, we obtain that

$$0\leq \int_{T}^{+\infty }\frac{-A∥\ddot{x}(s){∥}^{2}-B(s)∥\dot{x}(s){∥}^{2}}{∥\ddot{x}(s)∥+p(s)∥\dot{x}(s)∥}\;ds\leq \phi (H(u(T),v(T))-H(\bar{x},\bar{x})),$$

which leads to

$$t\mapsto \frac{∥\ddot{x}(t){∥}^{2}}{∥\ddot{x}(t)∥+p(t)∥\dot{x}(t)∥},\;t\mapsto \frac{∥\dot{x}(t){∥}^{2}}{∥\ddot{x}(t)∥+p(t)∥\dot{x}(t)∥}\in L^{1}([t_{0},+\infty ),R^{n})$$

and further to

$$t\mapsto \frac{∥\ddot{x}(t)∥∥\dot{x}(t)∥}{∥\ddot{x}(t)∥+p(t)∥\dot{x}(t)∥}\in L^{1}([t_{0},+\infty ),R^{n}).$$

Since *p* is bounded from above on $\left[t_{0},+\infty \right)$ and

$$∥\dot{x}(t)∥+∥\ddot{x}(t)∥=\frac{∥\ddot{x}(t){∥}^{2}}{∥\ddot{x}(t)∥+p(t)∥\dot{x}(t)∥}+\frac{p(t)∥\dot{x}(t){∥}^{2}}{∥\ddot{x}(t)∥+p(t)∥\dot{x}(t)∥}+\frac{(1+p(t))∥\ddot{x}(t)∥∥\dot{x}(t)∥}{∥\ddot{x}(t)∥+p(t)∥\dot{x}(t)∥},$$

we obtain that

$$\dot{x},\ddot{x}\in L^{1}([t_{0},+\infty ),R^{n}).$$

Finally, since $\dot{x}\in L^{1}([t_{0},+\infty ),R^{n})$, the limit $\lim_{t⟶+\infty }x(t)$ exists and it is finite. In conclusion, there exists $\bar{x}\in \mathrm{crit}(g)$ such that

$$\lim_{t⟶+\infty }x(t)=\bar{x}.$$

Remark 3.2 According to Remark 2.1, there exists *N*>0 such that $∥x^{\left(3\right)}(t)∥\leq N(∥\ddot{x}(t)∥+∥\dot{x}(t)∥)$ for almost every $t\geq t_{0},$ hence, under the hypotheses of Theorem 3.2, one has

$$x^{\left(3\right)}\in L^{1}([t_{0},+\infty ),R^{n}).$$

Remark 3.3 Since the class of semi-algebraic functions is closed under addition [see, for example, [27]] and $(x,y)\mapsto \frac{1}{2}∥x-y{∥}^{2}$ is semi-algebraic, the conclusion of the previous theorem holds, if, instead of asking that *H* is a KL function, we ask that *g* is semi-algebraic.

Remark 3.4 Assume that *g* is coercive, that is

$$\lim_{∥u∥\to +\infty }g(u)=+\infty .$$

For $u_{0},v_{0}\in R^{n}$, let $x\in C^{2}([0,+\infty ),R^{n})$ be the unique global solution of (2). Then *x* is bounded.

Indeed, notice that *g* is bounded from below, being a continuous and coercive function [see [28]]. From (10), it follows that $\beta \dot{x}(T)+x(T)$ is contained for every $T\geq t_{1}$ in a lower level set of *g*, which is bounded. According to Theorem 3.1, $\beta \dot{x}(t)⟶0,\;t⟶+\infty$, which implies that *x* is bounded.

## 4. Convergence rates

In this section, we will assume that the regularized function *H* satisfies the Lojasiewicz property, which, as noted in the previous section, corresponds to a particular choice of the desingularizing function *ϕ* [see [19,22,25]].

Definition 4.1 Let $f:R^{n}⟶R$ be a differentiable function. The function *f* is said to fulfil the Łojasiewicz property, if for every $\bar{x}\in \mathrm{crit}\;f$ there exist K,ϵ>0 and θ∈(0,1) such that

$$|f(x)-f(\bar{x}){|}^{\theta }\leq K∥\nabla f(x)∥\text{ for every }x\text{ fulfilling }∥x-\bar{x}∥<\epsilon .$$

The number *θ* is called the Łojasiewicz exponent of *f* at the critical point $\bar{x}.$

In the following theorem, we provide convergence rates for the trajectory generated by (2), its velocity and acceleration in terms of the Łojasiewicz exponent of *H* [see, also, [22,25]].

Theorem 4.1 Assume that *g* is bounded from below and, for $u_{0},v_{0}\in R^{n},$ let *x* be the unique strong global solution of (2). Suppose that *x* is bounded, let $\bar{x}\in \mathrm{crit}(g)$ be such that $\lim_{t⟶+\infty }x(t)=\bar{x}$ and suppose that

$$H:R^{n}\times R^{n}⟶R,\;H(x,y)=g(x)+\frac{1}{2}∥x-y{∥}^{2}$$

fulfils the Łojasiewicz property at $(\bar{x},\bar{x})\in \mathrm{crit}\;H$ with Łojasiewicz exponent *θ*. Let be (see Remark 2.1)

$$N:=\max_{t\geq t_{0}}\left(L_{g}+\frac{|\alpha |}{t^{2}},\;\gamma +\frac{|\alpha |}{t}\right).$$

Then, there exist $a_{1},a_{2},a_{3},a_{4}>0$ and *T*>0 such that for almost every t∈[T,+∞), the following statements are true:

1. if $\theta \in (0,\frac{1}{2}),$ then *x* converges in finite time;
2. if $\theta =\frac{1}{2},$ then $∥x(t)-\bar{x}∥\leq a_{1}e^{-a_{2}t},$ $∥\dot{x}(t)∥\leq a_{1}e^{-a_{2}t}$ and $∥\ddot{x}(t)∥\leq Na_{1}e^{-a_{2}t};$
3. if $\theta \in (\frac{1}{2},1),$ then $∥x(t)-\bar{x}∥\leq (a_{3}t+a_{4}{)}^{-(1-\theta )/(2\theta -1)},$ $∥\dot{x}(t)∥\leq (a_{3}t+a_{4}{)}^{-(1-\theta )/(2\theta -1)}$ and $∥\ddot{x}(t)∥\leq N(a_{3}t+a_{4}{)}^{-(1-\theta )/(2\theta -1)}.$

Proof. Let be $0<\beta <\min(2/L_{g},(\sqrt{L_{g}^{2}+2\gamma L_{g}}-L_{g})/L_{g})$ and $t_{1}>0$ such that for every $t\geq t_{1}$ the inequalities (9) hold. We define for every $t\in [t_{1},+\infty )$

$$\sigma (t):=\int_{t}^{+\infty }(∥\dot{x}(s)∥+∥\ddot{x}(s)∥)\;ds.$$

Let $t\in [t_{1},+\infty )$ be fixed. For T≥t, we have

$$∥x(t)-\bar{x}∥=∥x(T)-\bar{x}-\int_{t}^{T}\dot{x}(s)\;ds∥\leq ∥x(T)-\bar{x}∥+\int_{t}^{T}∥\dot{x}(s)∥\;ds.$$

By taking the limit as T⟶+∞, we obtain

(15) $$∥x(t)-\bar{x}∥\leq \int_{t}^{+\infty }∥\dot{x}(s)∥\;ds\leq \sigma (t).$$

Furthermore, for *T*>*t*, we have

$$∥\dot{x}(t)∥=∥\dot{x}(T)-\int_{t}^{T}\ddot{x}(s)\;ds∥\leq ∥\dot{x}(T)∥+\int_{t}^{T}∥\ddot{x}(s)∥\;ds.$$

By taking the limit as T⟶+∞, we obtain

(16) $$∥\dot{x}(t)∥\leq \int_{t}^{+\infty }∥\ddot{x}(s)∥\;ds\leq \sigma (t).$$

According to Remark 2.1, it holds $∥x^{\left(3\right)}(t)∥\leq N(∥\ddot{x}(t)∥+∥\dot{x}(t)∥)$ for almost every $t\geq t_{1}$,

$$\begin{array}{rl} ∥\ddot{x}(t)∥ & =∥\ddot{x}(T)-\int_{t}^{T}x^{\left(3\right)}(s)\;ds∥\leq ∥\ddot{x}(T)∥+\int_{t}^{T}∥x^{\left(3\right)}(s)∥\;ds\leq ∥\ddot{x}(T)∥\\ & \;+N\int_{t}^{T}(∥\ddot{x}(s)∥+∥\dot{x}(s)∥)\;ds\;\forall \;T>t. \end{array}$$

By taking the limit as T⟶+∞, we obtain

(17) $$∥\ddot{x}(t)∥\leq N\sigma (t).$$

Next, we show that there exists *m*<0 such that

(18) $$\frac{A∥\ddot{x}(t){∥}^{2}+B(t)∥\dot{x}(t){∥}^{2}}{∥\ddot{x}(t)∥+p(t)∥\dot{x}(t)∥}\leq m(∥\dot{x}(t)∥+∥\ddot{x}(t)∥).$$

Indeed,

$$\begin{array}{rl} & (∥\ddot{x}(t)∥+p(t)∥\dot{x}(t)∥)(∥\dot{x}(t)∥+∥\ddot{x}(t)∥)=∥\ddot{x}(t){∥}^{2}+(1+p(t))∥\dot{x}(t)∥∥\ddot{x}(t)∥+p(t)∥\dot{x}(t){∥}^{2}\\ & \;\leq \left(\frac{3}{2}+\frac{p(t)}{2}\right)∥\ddot{x}(t){∥}^{2}+\left(\frac{1}{2}+\frac{3p(t)}{2}\right)∥\dot{x}(t){∥}^{2}\leq \frac{A}{m}∥\ddot{x}(t){∥}^{2}+\frac{B(t)}{m}∥\dot{x}(t){∥}^{2}, \end{array}$$

where

$$m:=\max\left(\max_{t\geq t_{1}}\frac{A}{3/2+p(t)/2},\max_{t\geq t_{1}}\frac{B(t)}{(3/2)p(t)+1/2}\right).$$

It is an easy verification that m<0.

As we have seen in the proof of Theorem 3.2, if there exists $\bar{t}\geq t_{1}$ such that $H(u(\bar{t}),v(\bar{t}))=H(\bar{x},\bar{x})$, then *x* is constant on $\left[\bar{t},+\infty \right)$ and the conclusion follows.

On the other hand, if for every $t\geq t_{1}$ one has that $H(u(t),v(t))>H(\bar{x},\bar{x})$, then according to the proof of Theorem 3.2, there exist ϵ>0 and $T\geq t_{1}$ such that

$$∥(u(t),v(t))-(\bar{x},\bar{x})∥<\epsilon$$

and

$$\frac{d}{dt}(H(u(t),v(t))-H(\bar{x},\bar{x}){)}^{1-\theta }\leq \frac{A∥\ddot{x}(t){∥}^{2}+B(t)∥\dot{x}(t){∥}^{2}}{∥\ddot{x}(t)∥+p(t)∥\dot{x}(t)∥}\text{ for almost every }t\geq T.$$

Busing (18), we obtain that

$$M(∥\dot{x}(t)∥+∥\ddot{x}(t)∥)+\frac{d}{dt}(H(u(t),v(t))-H(\bar{x},\bar{x}){)}^{1-\theta }\leq 0\text{ for almost every }t\geq T,$$

where M:=−m>0.

For t≥T, we integrate the last relation on the interval $\left[t,\tilde{T}\right]$, where $\tilde{T}>t$, which yields

$$M\int_{t}^{\tilde{T}}(∥\dot{x}(s)∥+∥\ddot{x}(s)∥)\;ds+(H(u(\tilde{T}),v(\tilde{T}))-H(\bar{x},\bar{x}){)}^{1-\theta }\leq (H(u(t),v(t))-H(\bar{x},\bar{x}){)}^{1-\theta }.$$

By taking the limits as $\tilde{T}⟶+\infty$, we get

$$M\sigma (t)\leq (H(u(t),v(t))-H(\bar{x},\bar{x}){)}^{1-\theta }.$$

On the other hand, according to the KL property for *H* and Lemma 3.3 (v), we have

$$(H(u(t),v(t))-H(\bar{x},\bar{x}){)}^{\theta }\leq K∥\nabla H(u(t),v(t))∥\leq K(∥\ddot{x}(t)∥+p(t)∥\dot{x}(t)∥)\text{ for almost every }t\geq T,$$

hence

$$M\sigma (t)\leq K^{(1-\theta )/\theta }(∥\ddot{x}(t)∥+p(t)∥\dot{x}(t)∥{)}^{(1-\theta )/\theta }\text{ for almost every }t\geq T.$$

By denoting $a:=\max_{t\geq T}p(t)\in (0,+\infty )$, one can easily see that *a*>1 and so

$$M\sigma (t)\leq (aK{)}^{(1-\theta )/\theta }(∥\dot{x}(t)∥+∥\ddot{x}(t)∥{)}^{(1-\theta )/\theta }\text{ for almost every }t\geq T.$$

Taking into account that $∥\dot{x}(t)∥+∥\ddot{x}(t)∥=-\dot{\sigma }(t)$, the previous inequality is nothing else than

(19) $$-c\sigma^{\theta /(1-\theta )}(t)\geq \dot{\sigma }(t)\text{ for almost every }t\geq T,$$

where $c:=M^{\theta /(1-\theta )}/aK>0.$

If $\theta =\frac{1}{2}$, then (19) becomes $c\sigma (t)+\dot{\sigma }(t)\leq 0\text{ for almost every }t\geq T.$ By multiplying with $e^{ct}$ and integrating on [T,t], it follows that there exists $a_{1}>0$ such that

$$\sigma (t)\leq a_{1}e^{-a_{2}t}\text{ for every }t\geq T,$$

where $a_{2}=c.$ Using (15)–(17), we obtain

$$∥x(t)-\bar{x}∥\leq a_{1}e^{-a_{2}t},∥\dot{x}(t)∥\leq a_{1}e^{-a_{2}t}\;\text{and}\;∥\ddot{x}(t)∥\leq Na_{1}e^{-a_{2}t}\text{ for every }t\geq T,$$

which proves (b).

Assume now that $0<\theta <\frac{1}{2}$. In this case, (19) leads to

$$\frac{d}{dt}\sigma^{\left(1-2\theta )/(1-\theta \right)}(t)=\frac{1-2\theta }{1-\theta }\sigma^{-\theta /(1-\theta )}(t)\dot{\sigma }(t)\leq -c\frac{1-2\theta }{1-\theta }\text{ for almost every }t\geq T.$$

By integrating on [T,t] we obtain

$$\sigma^{\left(1-2\theta )/(1-\theta \right)}(t)\leq -\bar{\alpha }t+\bar{\beta },\text{ for every }t\geq T,$$

where $\bar{\alpha }>0.$ Then there exists $\hat{T}\geq T$ such that σ(t)≤0 for every $t\geq \hat{T}$, thus, *x* is constant on $\left[\hat{T},+\infty \right)$ and (a) follows.

Assume now that $\frac{1}{2}<\theta <1.$ In this case, (19) leads to

$$\frac{d}{dt}\sigma^{\left(1-2\theta )/(1-\theta \right)}(t)=\frac{1-2\theta }{1-\theta }\sigma^{-\theta /(1-\theta )}(t)\dot{\sigma }(t)\geq c\frac{2\theta -1}{1-\theta }\text{ for almost every }t\geq T.$$

By integrating on [T,t] we obtain

$$\sigma^{\left(1-2\theta )/(1-\theta \right)}(t)\geq a_{3}t+a_{4}\text{ for every }t\geq T,$$

where $a_{3},a_{4}>0,$ or, equivalently,

$$\sigma (t)\leq (a_{3}t+a_{4}{)}^{-(1-\theta )/(2\theta -1)}\text{ for every }t\geq T.$$

Using again (15)–(17), we obtain

$$\begin{array}{rl} & ∥x(t)-\bar{x}∥\leq (a_{3}t+a_{4}{)}^{-(1-\theta )/(2\theta -1)},\;∥\dot{x}(t)∥\leq (a_{3}t+a_{4}{)}^{-(1-\theta )/(2\theta -1)}\;\text{and}\\ & \;∥\ddot{x}(t)∥\leq N(a_{3}t+a_{4}{)}^{-(1-\theta )/(2\theta -1)}\text{ for every }t\geq T, \end{array}$$

which proves (c).
